# Evaluating person-centered care in neurological outpatient care: a mixed-methods content validity study

**DOI:** 10.1186/s12912-024-01837-9

**Published:** 2024-03-25

**Authors:** Mia Olsson, Sidona-Valentina Bala, Peter Hagell

**Affiliations:** 1 Department of Medicine, Helsingborg Central Hospital, Helsingborg, Sweden; 2 Department of Health Sciences, Faculty of Medicine, Lund University, Lund, Sweden; 3 The PRO-CARE Group, Faculty of Health Sciences, Kristianstad University, Kristianstad, SE-291 88 Sweden; 4 Restorative Parkinson Unit, Division of Neurology, Department of Clinical Sciences Lund, Lund University, Lund, Sweden

**Keywords:** Evaluation, Multiple sclerosis, Neurology, Outpatient care, Parkinson’s disease, Person-centered care

## Abstract

**Background:**

Person-centered care (PCC) is gaining increased attention. PCC concerns the whole person behind the disease and can improve care for people with long-term conditions such as multiple sclerosis (MS) and Parkinson’s disease (PD). However, there is a lack of tools to assess PCC from the patients’ perspective, particularly in outpatient care. The Person-Centered Care instrument for outpatient care (PCCoc) is an instrument under development with the intention to fill this gap. The aim of this study was to test the user-friendliness and content validity of the PCCoc as experienced by persons with MS and PD in neurological outpatient care.

**Methods:**

Twenty persons with MS or PD completed the 35-item PCCoc followed by an interview regarding the instrument’s intelligibility and ease of use to assess its user-friendliness. Participants then rated the relevance of each item. These ratings were used to calculate the content validity index (CVI) for individual items (I-CVI) and for the overall scale (S-CVI).

**Results:**

It took a median of 5 min for participants to complete the PCCoc. Instrument instructions were found clear, items easy to understand, and response categories distinct. No important missing areas were reported. I-CVI values ranged between 0.75 and 1, and S-CVI was 0.96.

**Conclusions:**

We found support for the user-friendliness and content validity of the PCCoc among persons with MS and PD, suggesting that the PCCoc can be useful for evaluating and developing PCC in neurological outpatient care. Further testing in broader contexts, including psychometric testing, is warranted to establish its usefulness.

## Introduction

There is no agreed upon general definition of person-centered care (PCC). However, its intention is that care should be informed by the individual’s thoughts, experiences, needs and preferences, and that patient interactions are empathetic and empowering [1]. The goal is to reach the person behind the disease and pay attention to both resources and barriers that may affect the person. Active listening, creating a partnership with the person, documentation of a care plan and shared decision-making are fundamental in PCC [2, 3].

In the last decades PCC has undergone considerable progress in areas such as inpatient and primary care but its assessment is still relatively underdeveloped, particularly in outpatient care [4–6], which is unfortunate since systematic assessment is an important aspect of quality assurance [1, 7]. According to De Boer et al. [8], PCC is important in the care of persons with all long-term conditions, regardless of diagnosis. An important goal in caring for persons with long-term conditions is to promote health despite disease and it is necessary to acknowledge every individual as a person to achieve this goal. A holistic view of the person and active involvement of family members have been identified as characteristics needed for successful care in long-term conditions [9]. However, the implementation of PCC in, e.g., neurological care appears to have been less marked than in other areas [10, 11].

Neurological disorders are the major cause of disability globally, and an increasing and ageing population will contribute to increasing needs for treatment, rehabilitation and nursing support in the future [12]. For example, globally it has been estimated that more than 2 million people were living with MS and 6.1 million individuals had PD in 2016, and that these numbers will increase over time [13, 14]. This is a large group of people that require individually adapted long-term care. Long-term neurological conditions such as MS and PD are characterized by a variety of signs and symptoms that affect both physical, cognitive, and emotional functions. Optic neuritis, limb weakness, sensory loss and fatigue are typical in MS as the chronic inflammatory disease affect the central nervous system [15]. In PD, motor symptoms of tremor, rigidity, bradykinesia, and postural instability are often accompanied by non-motor symptoms like depression, cognitive decline, sleep disorders and dysautonomia [16]. Both MS and PD are incurable, but several disease modifying drugs are available for MS, whereas PD therapy is symptomatic and mainly based on dopamine substituting medication [15, 16].

Given the importance of PCC and that long-term conditions primarily are managed in outpatient care, instruments that evaluate PCC in outpatient care are needed for the evaluation, development and future progress of PCC in the care of people with long-term disorders [5, 17, 18]. Patient-reported experience measures (PREMs) are tools intended to capture patients’ experiences of health care encounters [6, 19]. As such, instruments that assess patient experiences in terms of PCC represent one type of emerging PREMs [20–22] that can be useful in facilitating and improving PCC [19, 22, 23]. For example, communication between the patient and the healthcare professional can deepen with use of person-centered questions and guide the care of persons with, e.g., complex neurological conditions [24].

The Person-Centered Care instrument for outpatient care in rheumatology (PCCoc/rheum) is a PREM that aims to reflect perceived levels of PCC in outpatient care from a patient perspective. It was developed and tested in nurse-led outpatient rheumatology clinics and is based on a theoretically derived conceptual framework consisting of five interrelated and equally important domains (communication, social environment, personalization, shared decision-making, and empowerment) that operationalize outpatient PCC [25, 26]. Personalization, shared decision-making and empowerment represent an overlapping hierarchical continuum from lower to higher levels of outpatient PCC, whereas communication and social environment are integrated across the continuum. Since it first was developed, modifications of the instrument have been initiated with the aim to develop a generic version applicable not only to nurse-led clinics, as well as to persons with a broader range of long-term conditions, including neurological conditions. This was done by rewording items to avoid reference to nurses or rheumatology. In addition, new items were added to capture a broader range of PCC. This generic version is called the Person-Centered Care instrument for outpatient care (PCCoc) and currently consists of 35 items with four ordered response categories (0 = completely disagree to 3 = completely agree).

The aim of this study was to test the user-friendliness and content validity of the PCCoc as experienced by persons with MS and PD in neurological outpatient care.

## Methods

### Design

The study was designed as a mixed-methods study, based on structured interviews and relevance ratings of questionnaire items.

### Sample

We aimed for a sample of about 20 persons with MS or PD who had regular contact with the neurology outpatient clinic at a mid-sized south Swedish hospital. Inclusion criteria were Swedish speaking persons aged 18 years or older with a clinical diagnosis of MS or PD and at least three documented contacts (including at least two visits) at the clinic during the past year. Purposive sampling was employed to achieve variation in age, sex, and disease duration. Exclusion criteria was severe cognitive decline or dementia.

### Data collection and procedures

Participants were recruited by two registered nurses (who also obtained consent for the study), and all data were collected by the first author (MO) following a regular clinic visit. All respondents completed the 35-item PCCoc individually in the presence of an interviewer and the time taken to complete the questionnaire was recorded as an indicator of respondent burden [27]. The interviewer also noted whether the interviewee appeared to experience any issues (e.g., hesitations and other non-verbal or verbal reactions) when completing the questionnaire. After completing the PCCoc, a verbal probing cognitive interview [28] was conducted regarding whether respondents experienced any problems understanding questionnaire instructions and items, if any items were difficult to respond to, if anything important was missing, and if response categories were found distinct and easy to use. In addition, potential issues observed while respondents completed the questionnaire were followed up by the interviewer. During the interview, respondents were asked to comment and motivate their responses. All comments were noted verbatim by the interviewer and reviewed by the respondents for accuracy. Background data regarding age, gender, education, disease duration, perceived health [29], perceived disease severity [30], for how long they had received care at the outpatient clinic, and the number of clinic visits during the past year were then collected. Finally, the content validity of the PCCoc was assessed from the participants’ perspective. Participants were thus asked to rate how relevant they found each PCCoc item according to four ordered response categories (”not relevant”, ”somewhat relevant”, ”quite relevant” or ”highly relevant”).

### Analyses

Data were analyzed descriptively using mean (SD), median (q1-q3) and n (%), as appropriate. Interviews were analyzed by compiling comments across interviews and items, and then summarizing them regarding their contents and frequencies [28].

Content validity from the perspective of persons with MS and PD was assessed by calculating the Content Validity Index (CVI) [31, 32]. That is, the CVI for each item (I-CVI) was expressed as the proportion of respondents rating the item as ”quite” or ”highly” relevant, and the overall scale CVI (S-CVI) was expressed as the average I-CVI value [32]. I-CVI values of ≥ 0.78 and an S-CVI value of ≥ 0.90 have been suggested to indicate excellent content validity [32]. To explore the relationship between responses to PCCoc items and ratings of item relevance, we calculated Spearman correlations between these scores.

All statistical analyses were performed using IBM SPSS version 27 (IBM Corp., Armonk, NY, USA) and Microsoft Excel (version 16.0 for Microsoft 365).

## Results

Twenty-four people were approached and four declined participation (one person with MS and three with PD) due to lack of time and/or fatigue. Of the 20 participants, 13 had MS and seven had PD. Twelve respondents were women (60%) and eight were men, and their mean (SD) age was 52 (16.2) years (Table 1).

**Table 1:** Sample characteristics (*n* = 20) ^a^

MS / PD diagnosis, *n* (%)	13 (65) / 7 (35)
Age (years)	48 (37.5-67.25; 31–81)
Men / women, n (%)	8 (40) / 12 (60)
Educational level, n (%)	
Comprehensive school	3 (15)
Upper secondary school	6 (30)
University	11 (55)
Disease duration (years)	4.5 (1.75–9.5; 1–15)
Perceived health ^b,c^	2 (2–3; 1–4)
Perceived disease severity, *n* (%) ^c^	
Mild	11 (55)
Moderate	7 (35)
Severe	1 (5)
Duration of care at the outpatient clinic (years)	4.2 (1.3–8.5; 0.5–15)
Number of outpatient clinic visits past year	9 (3–13; 2–14)

^a^ Data are median (q1-q3; min-max) unless otherwise noted

^b^ Rated as Poor (0), Fair (1), Good (2), Very good (3) or Excellent (4)

^c^ Missing data for 1 person (5%)

MS, multiple sclerosis; PD, Parkinson’s disease

The median time taken to complete the PCCoc was 5 (q1-q3, 4.25–6.75; min-max, 4–12) minutes (mean (SD), 6 (2.2) minutes). The PCCoc was considered user-friendly and appealing by all respondents. Participants found the instructions clear (*n* = 18; 2 persons did not read the instructions) and items easy to understand (*n* = 16) and respond to (*n* = 14). Most respondents did not report that anything important was missing (*n* = 19), and response categories were found distinct (*n* = 19) and easy to use (*n* = 14). Participants commented that items were relevant to describe their care and that they were directed to the individual as a person.

A few participants found one or more items difficult to understand (*n* = 4) or respond to (*n* = 6). This primarily concerned items addressing family members, documentation, and written care plans, and was due to unclear wording, family members not being involved in their care, and lack of knowledge regarding their documentation/care plans. However, although difficult to respond to, these items were considered important. One person expressed concerns regarding the exact meaning of planning, decisions and implementation of care, and another person missed an item regarding available choices in one’s care.

Some respondents (n = 6) found minor difficulties in using the four response categories. The most common comments were that a “don’t know” category would have eased responding to some items, and that they missed a space for respondents to provide written comments. One person preferred a dichotomous response format (“agree”/”disagree”), while another wanted more response categories.

I-CVI values ranged between 0.75 and 1, and S-CVI was 0.96. Items 1 and 32 had the lowest I-CVI values (0.75); the remaining 33 items displayed values of 0.80 or higher (Table 2). The median number of items considered relevant by the participants was 34 (q1-q3, 33–35; min-max, 27–35). The mean Spearman correlation between PCCoc item scores and relevance ratings was 0.04 and ranged between − 0.65 and 0.66. Table 2 also reports I-CVI and S-CVI values based on the ratings by persons with MS and PD separately.

**Table 2:** Content validity index for individual PCCoc items and the overall scale ^a^

PCCoc items			I-CVI		
No.	Content (abridged)		All (*n* = 20)	MS (*n* = 13)	PD (*n* = 7)
1	Welcoming care environment		**0.75**	**0.77**	**0.71**
2	Undisturbed conversations		0.90	0.85	1.00
3	Equality in meeting		1.00	1.00	1.00
4	Confirmed as a person		1.00	1.00	1.00
5	Opportunity to tell my story		1.00	1.00	1.00
6	Understanding my situation		0.95	0.92	1.00
7	Experiences are respected		0.95	0.92	1.00
8	Self-knowledge is considered		0.95	0.92	1.00
9	Problems are taken seriously		1.00	1.00	1.00
10	Needs determine care planning		1.00	1.00	1.00
11	Agree with HCP on what to do		1.00	1.00	1.00
12	Gain new knowledge		0.80	**0.69**	1.00
13	Strengthened ability to cope		1.00	1.00	1.00
14	Coordinated care		1.00	1.00	1.00
15	Family participation		0.80	**0.77**	0.86
16	Care follow-up and documentation		1.00	1.00	1.00
17	Care responsibility is clear		0.95	0.92	1.00
18	Confident HCP contacts		1.00	1.00	1.00
19	Sufficient time allocated		1.00	1.00	1.00
20	Good HCP collaboration		1.00	1.00	1.00
21	Information faciliating decisions		1.00	1.00	1.00
22	Can influence care		1.00	1.00	1.00
23	Personal information documented		1.00	1.00	1.00
24	Care information shared as needed		1.00	1.00	1.00
25 ^b^	Active participation in care		0.95	0.92	1.00
26 ^b^	Encouraged to participate		1.00	1.00	1.00
27 ^b^	Involved in care		1.00	1.00	1.00
28 ^b^	Participate in care planning		1.00	1.00	1.00
29 ^b^	Participate in decisions on care		1.00	1.00	1.00
30 ^b^	Participate in implementing care		1.00	1.00	1.00
31 ^b^	Agreed written care plan		0.85	**0.77**	1.00
32 ^b^	Support for family members		**0.75**	**0.77**	**0.71**
33 ^b^	Achieve care goals		1.00	1.00	1.00
34 ^b^	Support to achieve care goals		1.00	1.00	1.00
35 ^b^	Own resources are utilized		0.95	0.92	1.00
S-CVI			0.96	0.95	0.98

^a^ I-CVI values below the suggested cut-off value (0.78) for excellent content validity [32] are bold

^b^ New items not included in the original PCCoc/rheum [25, 26]

I-CVI, item level content validity index; S-CVI, scale level content validity index; PCCoc, the person-centered care instrument for outpatient care; MS, multiple sclerosis; PD, Parkinson’s disease; HCP, health care professional(s)

## Discussion

This study provides support for the user-friendliness and content validity of the PCCoc from the perspective of persons with MS and PD in neurological outpatient care.

It took an average of less than 10 min to complete the PCCoc, which indicates an acceptable respondent burden [27]. In further support for this, items and response categories were in general considered easy to understand and use. This is an important aspect to consider, not least because fatigue is a common problem in a range of neurological and other long-term conditions, including MS and PD [33].

Respondent comments on the PCCoc were positive and they found its content relevant and without any important missing areas. Overall, instructions and items were found clear, easy to understand and respond to, and response categories were considered distinct. This is in close accordance with results from the original PCCoc/rheum in rheumatological nurse-led outpatient care where, e.g., 77% and 97% of respondents found items easy to understand and response categories distinct, respectively [25].

A few respondents would have preferred fewer or more response categories and some proposed a “don’t know” category. During the development of the original PCCoc/rheum, both two- and four-category response scales were tested and considered acceptable, although most participants preferred the four-category format [25]. While an extension into more than four response categories may be feasible, any additional category will need to be clearly distinct from the others and logically ordered to ensure that it works as intended. Furthermore, the use of neutral response categories such as “don’t know” are generally discouraged since they tend to be difficult to use and do not function as intended in practice [34].

One participant missed an item regarding available choices. While the relevance of this comment is acknowledged, it can also be considered covered by available items addressing, e.g., involvement and participation in care. The PCCoc exhibited excellent overall patient perceived content validity, with an S-CVI value above the suggested 0.9 cut-off [32]. With few exceptions, this was the case also at the individual item level, where all but a few items surpassed the suggested I-CVI cut-off of 0.78 [32]. However, it should be noted that this threshold concerns excellent content validity and that items with suboptimal values in this study approached the 0.78 threshold. Nevertheless, together with participants’ comments, these observations suggest that some items may benefit from further review. Importantly, our findings are in close accordance with those from the original PCCoc/rheum [25], which argues in favor of the generic nature of the concept and contents of the PCCoc. However, further studies in additional outpatient contexts are warranted to establish this property more firmly. Further refinement and testing of the PCCoc appears timely and warranted given the prominent role of outpatient care for people with long-term disorders and the lack of tools to evaluate PCC from a patient perspective in outpatient care [5, 6, 35]. Indeed, successful implementation of PCC requires direct patient input, which in turn requires PREMs that allow for systematic evaluation of the quality of care [6, 24].

Correlations between PCCoc item scores and relevance ratings were generally low and covered negative as well as positive associations of about the same size. Although not commonly addressed, this is relevant because it may be conceivable that a person’s experience may influence her or his relevance ratings. The lack of systematic associations and the occurrence of about equally sized negative as well as positive correlations strengthen our results and suggest that participants provided relevance ratings that were independent of their personal experiences.

Recent years have seen the appearance of various tools to assess PCC from a patient perspective [20–22]. However, in contrast to the PCCoc, these either lack an underpinning defined conceptual framework, derive their contents (items) from questionnaires developed for other purposes, or are not specifically targeting outpatient care. The PCCoc is based on the PCCoc/rheum, which was developed for rheumatological nurse-led outpatient care and is now being revised to be applicable also in other long-term conditions as well as across health care professionals. As such, the PCCoc is intended for use in quality assurance and monitoring at the level of outpatient units, as well as for follow-up of specific PCC implementation efforts, primarily through incorporation in web-based systems such as national quality registers [19, 23].

This study has limitations. For example, it may be argued that the sample was small. However, for studies of this kind samples of about the size used in here, or smaller, are recommended [31, 36, 37]. It may also be argued that assessment of content validity should be made with experts rather than patients. However, with patient-reported instruments intended to reflect patient experiences, it can also be argued that patients are the experts. Furthermore, PCCoc items were developed based on a conceptual framework for PCC in outpatient care [25], and the objective of this study was to assess the instrument’s user-friendliness and content validity from the perspective of persons with MS and PD. It is further acknowledged that respondent burden was assessed indirectly by means of the time taken to complete the PCCoc. Although this is a commonly used approach to address respondent burden, burden is a complex issue and requires a more comprehensive assessment to allow for firm conclusions [38, 39]. Finally, we did not test the psychometric properties of the PCCoc, which will be necessary before recommending its use. However, instrument evaluation from the respondents’ perspective is considered a pivotal prerequisite before undertaking larger scale psychometric testing [36, 37].

## Conclusion

Our findings support the user-friendliness and content validity of the PCCoc for capturing the experience of PCC in outpatient care for persons with MS and PD. Thus, the PCCoc shows promise as a tool for evaluating and developing PCC in neurological outpatient care. Further studies in broader neurological contexts as well as in outpatient care for people with other long-term conditions, including testing of its psychometric properties, are warranted to establish its usefulness more firmly.

## Data Availability

The datasets used and/or analysed during the current study are available from the corresponding author on reasonable request.

## Abbreviations

CVI: Content validity index
I-CVI: Item level content validity index
MS: Multiple sclerosis
PCC: Person-centered care
PCCoc: Person-Centered Care instrument for outpatient care
PCCoc/rheum: Person-Centered Care instrument for outpatient care in rheumatology
PD: Parkinson’s disease
S-CVI: Scale level content validity index
